# Tumour-derived extracellular vesicle based vaccines for melanoma treatment

**DOI:** 10.1007/s13346-023-01328-5

**Published:** 2023-04-06

**Authors:** Lorena Gonzalez-Melero, Rosa Maria Hernandez, Edorta Santos-Vizcaino, Manoli Igartua

**Affiliations:** 1 NanoBioCel Research Group, Laboratory of Pharmaceutics, School of Pharmacy, University of the Basque Country (UPV/EHU), Vitoria-Gasteiz, Spain; 2 Biomedical Research Networking Centre in Bioengineering, Biomaterials and Nanomedicine (CIBER-BBN), Institute of Health Carlos III, Madrid, Spain; 3 Bioaraba, NanoBioCel Research Group, Vitoria-Gasteiz, Spain

**Keywords:** Melanoma, Extracellular vesicles, Exosomes, Immunotherapy, Immunostimulatory molecules, Tumour cells

## Abstract

The interest of extracellular vesicles (EVs) in cancer immunotherapy is increasing every day. EVs are lipid bilayer vesicles released by most cells, which contain the molecular signature of their parent cell. Melanoma-derived EVs present antigens specific to this aggressive type of cancer, but they also exert immunomodulatory and pro-metastatic activity. Until now, most reviews focus on the immunoevasive characteristics of tumour-derived EVs, but do not help to overcome the issues related to them. In this review, we describe isolation methods of EVs from melanoma patients and most interesting markers to oversee their effect if they are used as antigen carriers. We also discuss the methods developed so far to overcome the lack of immunogenicity of melanoma-derived EVs, which includes EV modification or adjuvant co-administration. In summary, we conclude that EVs can be an interesting antigen source for immunotherapy development once EV obtaining is optimised and the understanding of the mechanisms behind their multiple effects is further understood.

**Graphical Abstract:**

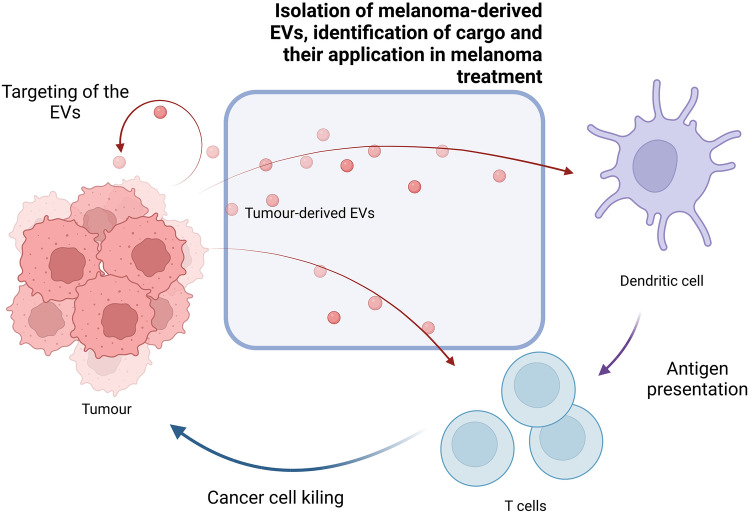

## Introduction

Melanoma is the most aggressive and deadliest type of skin cancer. Worldwide, melanoma represents 1.7% of all newly diagnosed cancers, and 0.6% of cancer related deaths [1]. In the last 70 years, melanoma incidence has increased in fair-skinned countries and is still far from been stable [2, 3]. On the contrary, mortality rates have not followed that pattern and have maintained stable in the last years [2, 3]. Early diagnosis and treatment improvement are responsible for the increased survival probability. In fact, 5-year survival probability is really high when diagnosed at an early stage and is still localised, but survival probability heavily drops with stage advancing to distant metastasised cancers; according to the National Cancer Institute, 5-year relative survival for the first case is 99.5% and 31.9% for the second one [4]. The most effective treatment for melanoma is surgery resection, and for unresectable metastatic melanoma, radiotherapy and chemotherapy have traditionally been used. These last two therapies, however, have shown many inconveniences like resistance, secondary cancers or toxicity to healthy tissues [5].

In recent years, two other therapies have emerged: targeted therapies and immunotherapy. Targeted therapies aim to combat molecular defects, but the majority of patients developed resistance in a short period of time [6]. Immunotherapy, on the other hand, helps patients’ immune system to restore its capacity to detect and eliminate cancer cells, avoiding the side effects related to the unspecificity caused by classical treatments. However, many patients do not respond to the treatment or resistances are developed [6]. In consequence, new immunotherapy strategies are under investigation. Ideally, the developed therapy should target the tumour without harming healthy cells and reprogram the microenvironment to re-enable immune system’s activity. In this context, new antigen sources that train immune cells for a precision therapy is heavily required.

Extracellular vesicles (EVs) are lipid bilayered vesicles released by a wide variety of cells into the extracellular space [7]. Currently, three main types of EVs can be distinguished based on their biogenesis, composition and cellular release: microvesicles, exosomes and apoptotic bodies [8]. Regardless their origin and cargo, all of them communicate with cells and modify microenvironments while they express the singularities of their progenitors [9, 10]. Apart from the main EV subtypes, there are other types of vesicles or fusions of them, like amphisomes, exomeres, ectosomes, or oncosomes [11, 12]. Additionally, melanoma cells secrete melanosomes, an EV-like structure involved in pigment transport to the outer layer of the skin [11]. The heterogeneity in EV population is as broad as their roles, and we have only scratched the surface so far. In recent years, the EV research area has flourished and provided extensive knowledge. Consequently, EV classification is in constant adaptation to match the development in this field of knowledge.

Due to their high metabolic activity, cancer cells release high numbers of EVs compared to any other cell type [13]. Because EVs are the reflection of the parent cell, depending on the cancer stage EVs function vary [13], which has made them increasingly interesting in cancer immunotherapy [9]. Their capacity to induce pro- and anti-tumour effects makes them the perfect tool to study and diagnostic cancer, evaluate treatment response and develop new therapies for cancer treatment [9, 10].

Independently of tumour stage, tumour-derived EVs are a source of tumour-associated antigens (TAA) and tumour-specific antigens (TSA), which T cells recognise and DCs cross-prime to activate cytotoxic T lymphocytes (CTLs) [13, 14]. Thus, direct tumour-derived EV vaccination shows a promising approach for cancer treatment. EVs have been found to be biocompatible with low or none toxicity [14]. Melanoma cell-derived EVs obtained from different origins (sera, melanoma tissue), as well as melanoma cell line derived ones are mentioned in this review. For treatment purposes, patient-derived EVs are the ideal ones; however, not always is possible to obtain enough quantity. Hence, until EV isolation techniques are optimised, vesicles produced from melanoma cell membranes have been developed, which are easy to produce and allow us to take advantage of the antigens expressed in tumour cells.

**Fig. 1:**
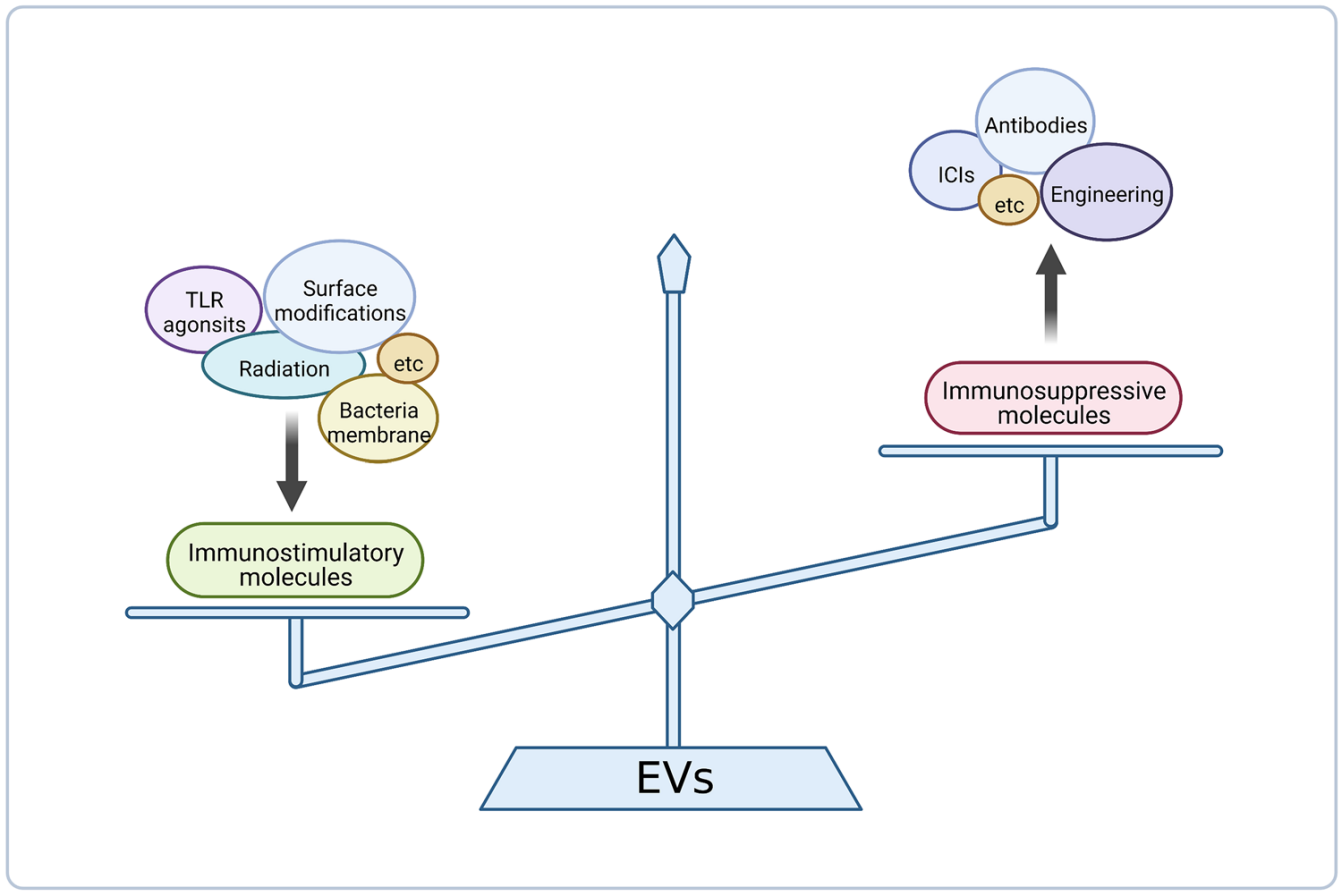
The duality in immune response of melanoma cell derived exosomes and the possible strategies to potentiate immunostimulatory abilities or reduce immunosuppressive ones. Among the strategies to potentiate immunostimulatory capacity, EV modification (such as surface modifications or cell radiation) or co-administration with adjuvants (like Toll-like receptor agonists (TLR) or bacteria membrane particles) are possible. On the other hand, immunosuppressive capacity can be obstructed blocking immunossupressive molecules with antibodies like immune checkpoint inhibitors (ICIs). Created with BioRender.com

At present, numerous studies both in vitro and in vivo have confirmed the utility of tumour-derived EVs as cancer vaccines. The main problem of tumour-derived EVs is the inhibitory effect they can cause. Cancer cells use EVs to induce immunosuppression in the tumour site as well as in other places that may turn into metastatic niches. How to block this inhibitory effect and potentiate immunostimulatory ones is the key to the development of efficient tumour-specific cancer vaccines (Fig. 1) [15]. Nevertheless, characteristics and markers of each EV subset are still to be clarified. Only then, the role in cellular communication and physiological processes for each EV subtype can be determined, and therefore, their behaviour modified in our favour. For this end, firstly, distinction and separation methods must be optimised [8, 15].

In this review, we will describe the melanoma EVs isolation techniques developed until now, with special focus on melanoma patient derived ones, as well as determine the molecules expressed in them and their function. After, non-modified, modified or stress derived melanoma EVs developed to treat melanoma will be discussed, as well as melanoma cell membrane derived vesicles.

## Melanoma EV isolation

The first obstacle for selecting the EVs of interest starts at the beginning of the process. At present, ultracentrifugation (UC) is the gold standard isolation method, but among the popular ones there are also density gradient centrifugation, ultrafiltration (UF), size exclusion chromatography (SEC), polymer-based precipitation and immunoaffinity (IA) capture-based techniques [8, 16].

It has been proved that vesicles derived from the serum of a cancer patient and those derived from the tumour in culture possess the same molecular profile and could be used as reference. Bardi et al. saw that extracellular vesicles derived from various human melanoma cell lines (A-375, SKMEL-28 and C-32) are bigger and have a more negative Z potential than those derived from human primary melanocytes [17]. Nevertheless, how to isolate them from melanoma patients is the question to be made.

**Fig. 2:**
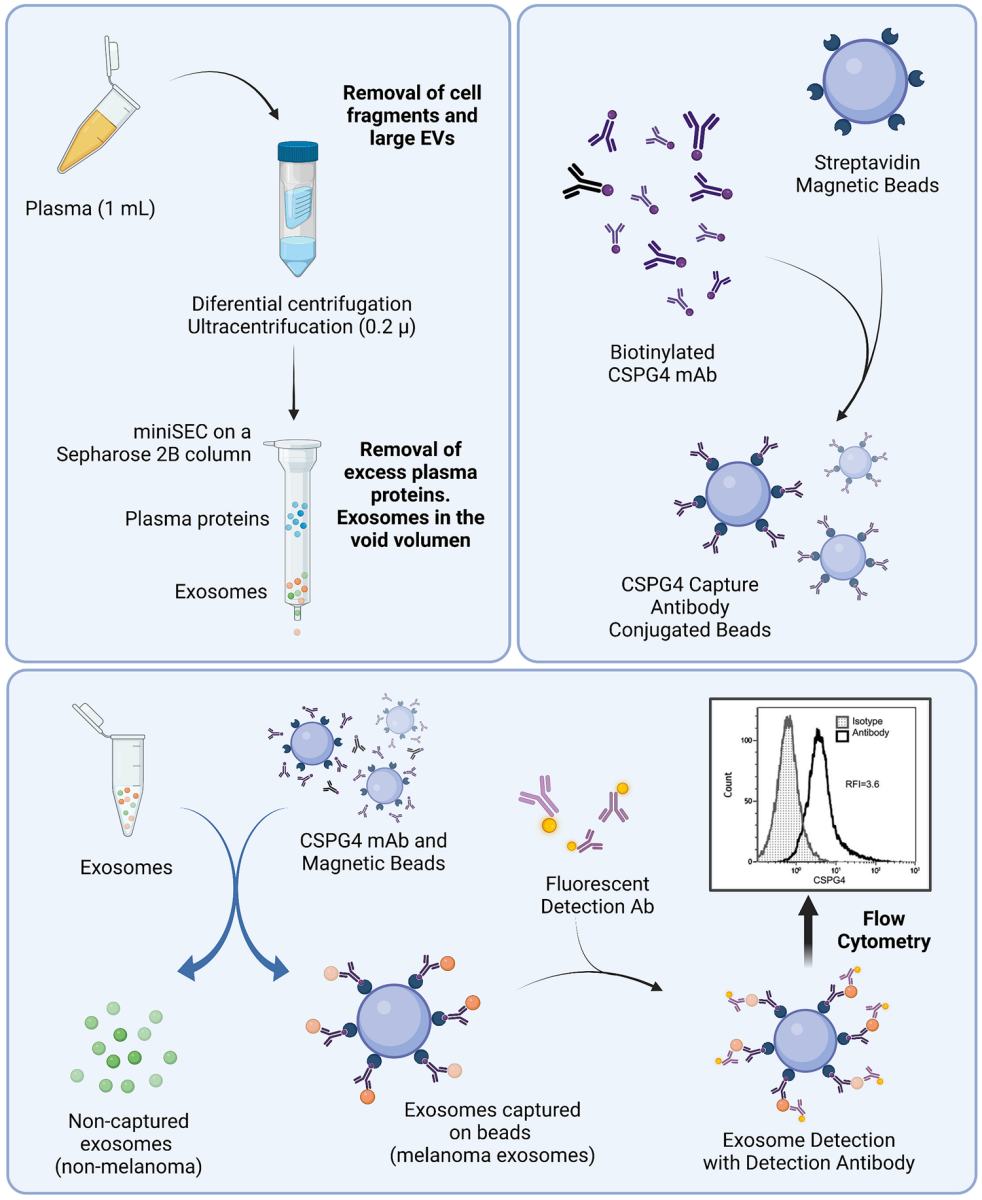
Melanoma cell-derived exosome isolation process. **a** A schema for isolation of exosomes from plasma. The recovered exosomes are partly “purified” by removal of protein/high density lipoproteins (HDL) complexes on the miniSEC column. **b** Exosomes are captured on streptavidin-labelled magnetic beads using pre-titered biotin-labelled anti-CSPG4 mAb. **c** Exosomes are co-incubated with biotinylated, pre-tittered anti-CSPG4 Ab and are captured on streptavidin-coated magnetic beads, and the bead-bound melanoma exosome-Ab complexes are recovered using a magnet. The antigen (CSPG4) carried by the bead-bound exosomes is detected using a fluorochrome-labelled and pre-tittered detection anti-CSPG4 mAb. The flow cytometry-based detection provides the relative fluorescence intensity (RFI) value for exosomes carrying the antigen. RFI = MFI of detection Ab/MFI of isotype control Ab. Non-captured exosomes in the supernatant are recovered as well for subsequent profiling by flow cytometry. Adapted from [18] and created with BioRender.com

In that regard, Sharma et al. separated plasma exosomes into melanoma and non-melanoma-derived exosomes by immunoaffinity-based isolation (Fig. 2). First, plasma exosomes were isolated by mini-size exclusion chromatography (miniSEC), and then separated by immune capture with CSPG4-specific monoclonal antibody (mAb). Chondroitin sulphate proteoglycan 4 (CSPG4) is a cancer-associated protein highly expressed in melanoma cells that plays a major role in tumour growth, migration and neo-angiogenesis [18]. That way, exosomes expressing CSPG4 were considered melanoma-derived exosomes.

**Fig. 3:**
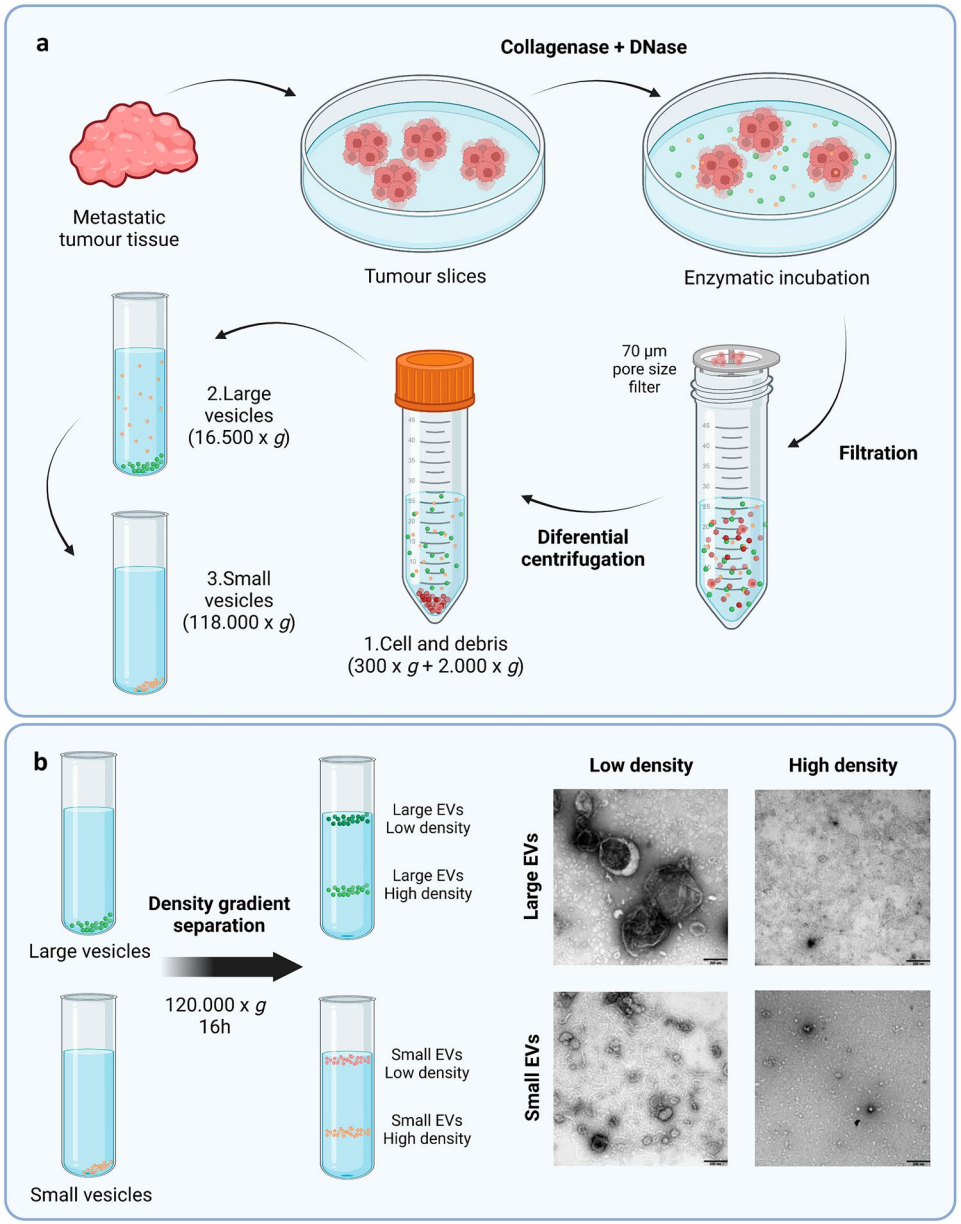
Schematic overview of the centrifugation-based protocol used to isolate vesicles from metastatic melanoma tissues, and posterior vesicle isolation through a density gradient separation. **a** The tumour tissues were dissected into smaller pieces that were incubated in cell culture media containing collagenase D and DNase I for 30 min allowing the tissue EVs to be released into the media. EVs were isolated from the media by differential centrifugation resulting in two subpopulations of EVs (large EVs (16,500 × *g*_avg_ pellet) and small EVs (118,000 × *g*_avg_ pellet)). **b** Isolated large and small EVs were bottom loaded on an iodixanol gradient (45–20%). From both populations of vesicles, two fractions were visible after the separation, with one fraction containing high density EVs (1.16–19 g/cm^3^) and one containing low density EVs (1.11–1.12 g/cm^3^). Ten micrograms of low and high density vesicles from both large and small EVs were loaded onto grids, negative stained and evaluated by transmission electron microscopy. Scale bars are 200 nm. Adapted from [19] and created with BioRender.com

On another approach, Crescitelli, R. et al. developed a technic to isolate EVs directly from melanoma tissues via enzymatic treatment, with the aim of obtaining EVs enriched in tumour-derived ones (Fig. 3). First, the tumour was dissected, and then, treated with DNase I and collagenase D for 30 min in culture media. Afterwards, the supernatant was collected, filtrated and centrifuged for cell and tissue debris elimination. The EVs obtained were first isolated by ultracentrifugation (size separation), and further separated by density gradient. The different fractions obtained were characterised regarding electron microscopy imaging, RNA profile and proteomics [19]. They have further optimised the protocol to obtain the EVs in 8 h [20].

However, these isolation methods do not enable an effective separation by cellular origin or even vesicle type, making it difficult to investigate the biological functions of each EV subtype [8]. For example, even though exosomes and microvesicles have different formation mechanisms, their size and density range overlap, and although exosomes are sorted by endosome-associated proteins (commonly CD9, CD63 and CD81), some microvesicles are sorted too because they express some of those endosomal proteins [16]. Thus, the resulting product is a mixture of many EV types and components of the biologic fluid of origin [8, 11, 16]. Moreover, even when EVs from one cell type culture medium are isolated, each isolation method leads to a different protein profile of EVs [8]. In short, a separation method that selectively isolates EV of interest is yet to be developed.

In summary, a combination of isolation methods is the best option for cancer cell-derived EV isolation [8, 16], until a more selective method is developed. However, we must take into account that all isolation methods influence EV purity and vesicle content; therefore, the isolation method selected for EV isolation should be carefully considered depending on our objectives [21], as well as tumour stage and experimental conditions for testing. In a few words, optimal isolation of EVs is the first challenge to their use in immunotherapy.

In the process, the characteristics of the EVs should be described. In this way, we will be able to know and gain leverage of the physicochemical and biochemical properties of our objective. There are two main types of analysis to characterise EVs: physical analysis and chemical/biochemical/compositional analysis. The first one includes, among others, nanoparticle tracking analysis (NTA), dynamic light scattering (DLS) and electron microscopy, and the second one includes immunodetection methods (like flow cytometry or western blotting) and proteomic analysis [8, 22]. With those technics, we should learn from our target cells and develop a more specific isolation method to avoid contamination with unwanted EVs.

## Melanoma molecules and immune response

Several studies have demonstrated the capacity of EVs for both suppressing and activating the immune response. Nevertheless, this phenomenon does not have to be contradictory by itself, as a wide variety of extracellular particles can be released by cancer cells, which, at the same time, can be in different cell states during tumour development. For this reason, depending on the EVs we isolate, the effect observed might be conditioned as the EVs can contain many types of functional molecules. In other words, the content of the EVs determines their function [23].

In late stage cancer, tumour cells develop immunevasive characteristics to escape from the immune system, and those factors that suppress the immune response are expressed in EVs as well. On the contrary, in the early stage of cancer, EVs express tumour antigens that are able to stimulate and activate the immune response. Nevertheless, this data variability may be related to the different experimental conditions used in each experiment, as well as the developmental stage of the cell of choice [24].

Altogether, tumour-derived EVs have a dual effect in the immune system. They can exert immunosuppressive or immunostimulatory effects, and, depending on the tumour stage, one effect or the other is enhanced [15].

Tumour-derived EVs carry a variety of biologically active molecules determined by the parent cell. Those molecules can be enzymes, growth factors, oncogenes and signalling immunoregulatory proteins [10]. Melanoma-derived vesicles are also a tumour antigen source with ability to induce epitope cross-presentation in dendritic cells [25] and even activate cytotoxic T-lymphocytes against them [13], but they have shown the capacity to support expansion of CD4^+^CD25^high^FOXP3^+^ regulatory T cells (Treg), while eliminating activated CD8 + antitumor effector cells [26]. This contradiction is because EVs act as reflections of the phenotypic change in progenitor cells. In consequence, the molecule profile they express enables us the monitoring of cancer progression [27, 28] and foresee their effect in immunotherapy.

**Fig. 4:**
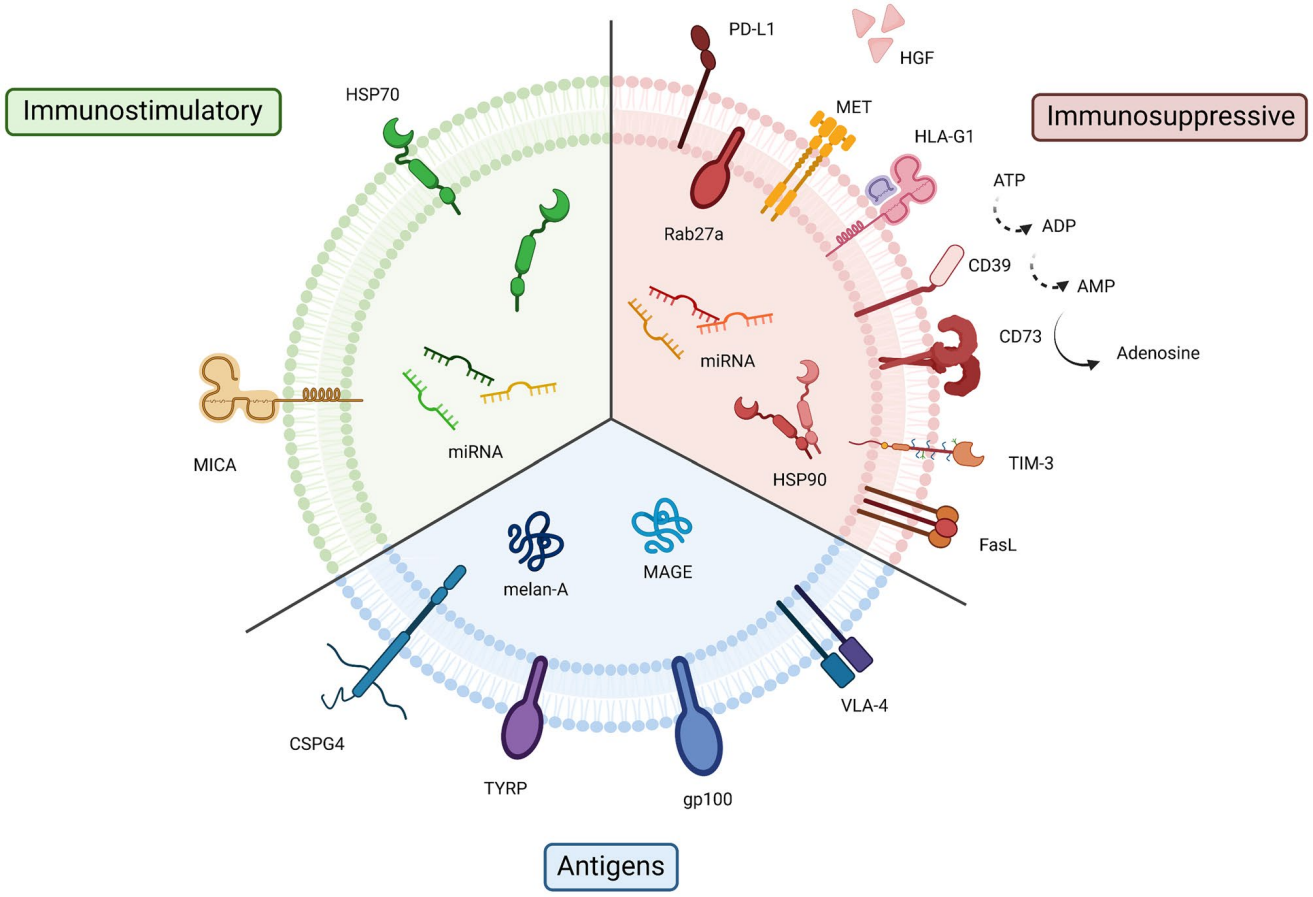
Molecules expressed in melanoma-derived EVs. Molecules expressed in melanoma-derived EVs. In green, immunostimulatory molecules are represented; in red, immunosuppressive molecules; and in blue, antigens recognised by immune system. Abbreviations: microRNA, miRNA; major histocompatibility complex class I chain-related protein A, MICA; heat shock protein 70 KDa, HSP70; programmed cell death ligand 1, PD-L1; mesenchymal-epithelial transition factor, MET; hepatocyte growth factor, HGF; non-classical human leucocyte antigen G class I, HLA-G1; cluster of differentiation, CD; T cell immunoglobulin and mucin domain-containing-3, TIM-3; Fas ligand, FasL; heat shock protein 90 KDa, HSP90; very late antigen 4, VLA-4; glycoprotein 100, gp100; tyrosinase-related protein, TYRP; chondroitin sulphate proteoglycan 4, CSPG4; melanoma antigen gene family, MAGE; melanocyte antigen, Melan-A; adenosine tri-di-monophosphate, ATP, ADP, AMP. Created with BioRender.com

The following sections aim to summarise the molecules expressed in melanoma-derived vesicles (Fig. 4), and their effect in immune response. Other kind of EVs that affect anti-tumour immunity have been reviewed somewhere else [29].

### Antigens expressed in melanoma cell-derived EVs

Tumour antigens have shown potential to activate T cells. There are two mayor tumour antigen groups: TAAs and TSAs. TSAs are mutated antigens only expressed in tumour cells, and TAAs are non-mutated antigens overexpressed or aberrantly expressed in tumour cells [30].

Antigens that appear in melanoma cells are released into EVs and have potential to activate the immune response against them. For example, melanoma exosomes derived from Fon and Mel-888 cell lines were able to deliver tumour antigens to dendritic cells (DCs), concretely melan-A/MART-1 (melanocyte antigen/melanoma-associated antigen recognised by T cells) and were able to activate CTLs [13]. For this end, the selected antigen must be expressed in greater quantity in melanoma-derived EVs than in normal cell-derived ones.

Melanoma antigens found in EVs are Melan-A/MART-1, glycoprotein 100 (gp100), tyrosinase-related protein (TYRP), melanoma antigen gene family (MAGE), very late antigen 4 (VLA-4) and CSPG4 (Fig. 4). Each of them is further expanded on Box 1. 

Most of the antigens presented are melanocyte differentiation antigens, which means they are associated with melanin production [39]. In that regard, differentiation antigens are normal non-mutated proteins, but they are overexpressed in melanoma.

In melanoma patients, exosomes found in ascites contained antigen-presenting molecules, as well as many tumour antigens like Melan-A/MART-1, TYRP or gp100, and were able to induce CTLs specific to Melan-A/MART-1 expansion through antigen deliver to DCs [25].

In the same way, Peinado et al. found that exosomes isolated from stage IV melanoma patients plasma contained, among others, TYRP-2 and VLA-4 [40].

In a posterior study, CSPG4, an epitope uniquely expressed on melanoma cells, was used to separate melanoma-derived exosomes from non-tumoral ones among serum exosomes (see the “Melanoma EV isolation” section) [18, 41]. CSPG4, also known as melanoma-associated chondroitin sulphate proteoglycan (MCSP) or neuron-glial antigen 2 (NG2), is a transmembrane proteoglycan upregulated in melanoma. Originally, it was described as highly immunogenic melanoma antigen, but later metastatic functions have been attributed to it [38]. Melanoma-derived exosomes isolated with anti-CSPG4 express various melanoma associated antigens: Melan-A/MART-1, gp100, VLA-4 and TYRP2, which are absent in non-melanoma exosomes [41].

### Immunostimulatory molecules in melanoma-derived EVs

As previously said, melanoma-derived EVs possess immunostimulatory molecules. Nevertheless, the interest of melanoma is to avoid immunological recognition. For the purpose of achieving immunosuppressive effects, EVs do not express as many immunostimulatory as immunomodulatory molecules [41].

Melanoma exosomes derived from non-metastatic cell lines and patients with non-metastatic primary melanomas trigger immune surveillance in pre-metastatic niches by NK cells and macrophages, causing cancer cell clearing [42]. However, when cancer stage advances, the molecular pattern changes, and with it, the activity of EVs.

Normally, regulators of the immune response are studied in melanoma-derived EVs in order to understand their role in cancer progression. If we want to use them in immunotherapy, the immunostimulatory capacity of EVs has to be studied, as well as how to potentiate it. In this subsection, immunostimulatory molecules present in melanoma-derived EVs will be reviewed (Fig. 4).

#### Proteins

HSP70 is a chaperon highly expressed in melanoma cells and their exosomes [40, 43]. As a chaperone, its function consists on ensuring correct protein function, but can directly inhibit apoptosis as well. In consequence, it is up-regulated in the presence of stress stimuli to avoid stress induced cell death [44]. In this context, HSP70 acquires the capacity to translocate to the extracellular milieu from the cytosol and has been found on the plasma membrane or associated with EVs [44–46].

Intracellular HPS70 in melanoma cells has showed pro-tumour activities such as avoiding programmed cell death or increasing cell growth rate. However, stress-induced (e.g. irradiation) HSP70 transport to the cell surface reverses its effects and triggers innate and specific immune responses; this is also achieved by exogenously administering HSP70 [43, 47]. HSP70 in tumour EVs acts as a damage-associated molecular pattern (DAMP) and consequently trigger anti-tumour immune responses. Additionally, it contributes to the cross-presentation of major histocompatibility complex (MHC) class I molecules [44, 48, 49], or even MHC-II [49] activating both CD8 + and CD4 + T cells. However, long-term exposure to HSP70 can induce immune tolerance depending on the cancer of origin [45]. In summary, HSP70 is involved in many facets of immunity and exhibits pro- or anti-tumour activities depending its location, expression level or origin [44]. Altogether, further research is needed to unveil physiopathological mechanisms related to HSP70 in melanoma.

Major histocompatibility complex (MHC) class I chain-related protein A (MICA) is a protein upregulated in stress, damage or transformation of cells, and ligand for immune receptor NKG2D (natural killer group 2D) that is expressed on human NK and CD8 + T cells [50]. In order to evade NKG2D mediated recognition, tumour cells shed MICA from their surface [51, 52], and this shedding from melanoma cells is achieved with exosomes [53]. Exosomal MICA molecules have been proved to downregulate NKG2D expression on circulating NK and CD8 + T cells, although the mechanism is unclear [54]. As a result, MICA containing exosomes suppress NK cytotoxicity, but, surprisingly, did not affect the activity of CTLs [54]. In consequence, high levels of NKG2D ligands in serum correlate with cancer stage and worse prognosis [51, 55]. In any case, immune recognition through NKG2D can be restored by maintaining MICA in tumour cell surface. For that end, many strategies that inhibit MICA shedding have been developed [51, 56], which resulted in maintenance of tumour recognition and arrest of tumour progression.

#### miRNA

miRNA is a non-coding RNA type with post-transcriptional gene expression regulator capacity. Depending on the miRNA, cancer development can be altered because they are able to stop cancer progression or to induce a more aggressive phenotype (Table 1).*miR* micro-RNA, *LIPA* lysosomal acid lipase A, *TAM* tumour associated macrophages, *TIMP3* tissue inhibitor of metalloproteinase 3, *MMP* matrix metalloprotein, *JAK2/STAT3* Janus kinase 2/signal transducer and activator of transcription 3, *SOCS1* suppressor of cytokine signaling 1, *CAF* cancer-associated fibroblasts, *OXPHOS* oxidative phosphorylation, *CTLs* cytotoxic T lymphocytes, *PI3K/AKT* phosphatidylinositol-3-kinase AKT*, TCR* T cell receptor, *(TNF-α)* tumour necrosis factor-alpha

**Table 1:** miRNA in melanoma-derived EVs that modify melanoma behaviour. miRNAs are listed by the order of presentation in the text.

Exosomal miRNA	Expression	Target cells	Function	Outcome	EV source	Ref.
miR-125b	↓	Tumour cells	c-Jun regulation and senescence inductor	Anti-apoptotic when decreased	Serum, primary melanoma and metastasis	[57–60]
miR-125b-5p	↑	Macrophages	Targets LIPA in macrophages	TAM formation and survival	Melanoma cell lines	[62]
miR-494	↑	Tumour cells	Cell apoptosis induction	miR-494 loss via exosomes promotes melanoma growth and cancer progression	Serum and A375 cells	[63]
miR-193a-3p and miR-193a-5p	↓	Tumour cells	Tumour cell viability suppressors	Down expression promotes cancer progression	Melanoma cell lines	[64, 65]
miR-21	↑	Fibroblasts	Downregulates TIMP3 and increases MMP	Fibroblast acquire aggressive phenotype	B16-F10 cells	[75]
miR-155-5p	↑	Fibroblasts	Activates JAK2/STAT3 pathway via SOCS1	Proangiogenic factors in CAFs	B16 and B16F10 cells	[76]
miR-155 and miR-210	↑	Fibroblasts	Aerobic glycolysis promotion and decreased OXPHOS	Acidification of the extracellular microenvironment	Melanoma cell lines	[77]
miR-192-5p	↑	CTLs	Targets ZEB2	CTLs cytotoxic activity suppression	Melanoma cell lines	[82]
let7 miRNAs (a, c, f), miR-31, miR34b and miR-185	↑	Tumour cells		Metastasis and invasion support	A375 cell line	[107]
miR-222	↑	Tumour cells	PI3K/AKT pathway induction	Promotes cell growth, migration and invasion and inhibits apoptosis	Melanoma cell lines	[108, 109]
miR-690	↑	CD4 + T cells	Activation of mitochondrial apoptotic pathway in CD4 + T cells	Promotes melanoma growth	B16F10 cell line	[110]
miR-3187-p, miR-498, miR149, miR-181a/b	↑	CD8 + T cells	TCR signalling and TNF-α secretion regulators	T cell suppression	Melanoma cell lines	[111]

Malignant melanoma cells reduce miR-125b expression because it is the post-transcriptional regulator of c-Jun, a positive regulator/inductor of tumour progression [57]. Melanoma cells treated with pre-miRNA-125b resulted in cell cycle arrest, and reduction in proliferation and in migration [57–59]. Therefore, miRNA-125b is known to be an inductor of melanoma cell senescence [58]. In exosomes, low levels of miRNA-125b correlate with advanced melanoma disease, as it presumably reflects dysregulation of tumor cells [60]. In uveal melanoma miRNA-125b-5p was also downregulated, probably due to its protective role [61]. However, miRNA-125b-5p has been detected in cutaneous melanoma-derived exosomes and exerted pro-tumour responses. Those exosomes showed the capacity to reprogram macrophages and to help in tumour-promoting tumour associated macrophage (TAM) phenotype formation and survival [62]. Hence, miRNA-125b modifies cell behaviour and the modulation outcome varies depending on the target cell.

Melanoma cell-derived exosomes are enriched in miR-494. Accumulation of miR-494 in melanoma cells suppresses the malignant phenotype by inducing cell apoptosis; to avoid that, melanoma cells get rid of miR-494 via exosome secretion [63].

On the contrary, melanoma cells downregulate miR-193a-3p and miR-193a-5p expression because they suppress growth and metastasis capacity. miR-193a-3p and -5p act as tumour suppressors in melanoma cells and are downregulated in melanoma-derived exosomes in patients’ plasma compared to healthy control ones. When melanoma cell lines were transfected with both miRNA-193a types, a down-modulation of pro-proliferative molecules and anti-apoptotic factors, among others, was detected, which results in anti-proliferative and pro-apoptotic signals [64]. Furthermore, miR-193a-3p and miR-193a-5p have also shown down-regulation capacity of programmed cell death ligand-1 (PD-L1), reducing the ability of melanoma cells to scape immune recognition [65].

### Immunosuppressive molecules and pro-tumour behaviours in melanoma-derived EVs

Melanoma-derived EVs induce mixed biological effects, from inducing anti-tumour response to tumour immune scape. Nevertheless, the balance tends to tip towards immunoevasion, and immune suppression mechanisms stand out [66]. This is because melanoma cell-derived EVs have proven to carry multiple inhibitory signals that jointly inhibit recipient immune cells [41].

For example, B16-F10 melanoma cell line derived exosomes induce M1 anti-tumour and M2 pro-tumour macrophage activation phenotype, but both can exert pro-tumour supportive functions [67]. They also remodel lymph nodes, where they interact with lymphatic endothelial cells and medullary sinus macrophages and impair their function; as a result, melanoma-derived exosomes induce apoptosis of antigen-specific CD8 + T cells and favour tumour progression [68].

Disease stage determines the molecular cargo of EVs, and thereby, their function/activity. In a study carried out by Lazar et al., various melanoma cell lines were evaluated to determine their proteome. Total protein of non-tumorigenic, tumorigenic and metastatic cell lines were similar and shared the majority of the proteins identified, but metastatic ones showed specific protein signature. Those proteins could help melanoma cells to increase motility, angiogenesis and modulate immune response [33]. For that reason, exosomes derived from a more aggressive type of melanoma transfer their metastatic properties to less aggressive ones [69] and increase their migratory capacity [33]. Moreover, they also mediate strong immunosuppression of primary human immune cells in vitro and in vivo [70] and educate bone marrow-derived dendritic cells (BMDC) to facilitate the formation of pre-metastatic niches [40].

Even though cells fight to avoid cancer development, eventually tumour progression happens and those same cells favour tumour progression. That is the case for dermal fibroblasts, which play a protective roll at the beginning of melanoma development. However, due to the constant stimulatory signal release from tumour cells, fibroblasts can turn into cancer-associated fibroblasts (CAF) [71]. Hu et al. proved that B16F0 melanoma cell line-secreted exosomes induce fibroblast NIH/3T3 cell transformation into CAF via transferring lncRNA Gm26809 in exosomes [72]. In turn, CAF secretory phenotype is altered to maintain tumour progression and metastasis [73], and support migration and invasion of melanoma cells [74]. Moreover, Wang et al. saw that melanoma-derived exosomes transfer miR-21 to fibroblasts. miR-21 downregulates tissue inhibitor of metalloproteinase 3 (TIMP3) expression and increases matrix metalloprotein (MMP) expression in fibroblasts, meaning the fibroblasts acquire an aggressive phenotype [75]. Moreover, Zhou et al. demonstrated that exosomal miR-155-5p is the responsible of inducing proangiogenic CAFs [76]. In addition, melanoma cells deliver not only miR-155 but also miR-210 to fibroblast in their exosomes, which promotes aerobic glycolysis and decrease oxidative phosphorylation (OXPHOS). In consequence, the extracellular microenvironment is acidified to favour tumour development and a pre-metastatic microenvironment [77].

Rapid cell proliferation and lack of oxygen in tumour site decreases the pH of the microenvironment. Acidic pH of the microenvironment influences melanoma cells to a more malignant phenotype and increases exosome secretion [78, 79]. Furthermore, low pH alters membrane biophysical characteristics, which increases exosome uptake by melanoma cells by fusion [80]. Those exosomes can mediate intercellular cross-talk to give migratory and invasive capacities to other cancer cells because they transfer metastatic exosomal proteins to cancer cells [79], but they also can influence cells of the immune system and downregulate them [81]. Extracellular vesicles secreted under hypoxia conditions carry hypoxia-responsive miRNA, such as miR-192-5p; the transfer of miR-192-5p to immune cells may suppress CTLs cytotoxic activity [82]. In summary, tumour development and progression is the result of a vicious circle of constant stimuli feedback.

Nevertheless, immunoinhibitory capacity is not just determined by the presence of immunohinibitory molecules in exosomes, but by the ratio of stimulatory/suppressor molecules in the EVs, and the melanoma-derived EVs/total EVs ratio in plasma [41]. In other words, immunosuppressive capacity of EVs is conditioned by the misbalance of immunosuppressive EVs and molecules. In melanoma, during tumour development the level of EVs in plasma is increased. Many of the EVs found in plasma have non-tumour origin and possess tumour-suppressive functions that seem to complement the anti-tumour immune response [83], so we just need to tip immunohinibitory–immunostimulatory scales in our favor.

In this section, immunosuppressive molecules expressed in melanoma-derived EVs are overviewed (Fig. 4).

#### Proteins

Fas (CD95) is a dead receptor present in the surface of various immune cells, and its activation induces cell apoptosis. The interaction with its ligand, FasL (CD95L), regulates various physiological processes, like the immune response, and is required for immune homeostasis [84, 85]. FasL present in melanoma-derived exosomes induces apoptosis of > 50% of activated T cells [41] and tumour reactive CTL [26].

Exosomal PD-L1 inhibits anti-tumour response by inhibiting CD8 + T cell proliferation and downregulating NKG2D receptor on NK cells [41]. Moreover, systemic exosomal PD-L1 supresses effector T lymphocytes even at a distant sites and enables tumour growth of those that cannot secrete their own [86]. PD-L1 expression in melanoma-derived exosomes is stimulated by interferon-γ (IFN-γ) and increases in response to anti-PD-1 immunotherapy. This is because when anti-PD-1 antibodies are used, T cell activity is restored and activated antigen-specific CD8 + T cells secrete IFN-γ. Taken together, exosomal PD-L1 increase is related to anti-PD-1 immunotherapy treatment [87, 88].

As well as PD-L1, TGF-beta pathway also downregulates NKG2D receptor on NK cells [41] and interferes with T cell activity in melanoma cell-derived exosomes. In particular, membrane-associated TGF-β present in exosomes impair the IL-2 induced proliferation of lymphocytes, only when CD4^+^ T-cell subset was present. In fact, IL-2 induces the expression of its ligand CD25 in lymphocytes and exosomes impair that expression in all but in CD3^+^CD8^−^ T cells [89]. Thus, TGF-β impairment is selective, because exosomes do not impair the response of CD4^+^CD25^+^ cells to IL-2, but rather promote their immunosuppressive phenotype and is thought to favour Treg expansion [26, 89]. TGF-β also inhibits the expression of effector molecules of CTL and NK, like perforin and granzymes [90].

CD73 is an ectonucleotidase expressed on melanoma cells and exosomes that catalyses extracellular adenosine [91]. Adenosine is a purine nucleoside responsible of many physio-pathological processes that are triggered in response to a distress like hypoxia or inflammation, among others. Its effects are mainly focused on tissue repair and protection, such as angiogenesis stimulation, extracellular matrix remodelling, or immune cell suppression [92]. However, in melanoma, its effects turn in tumour growth, immune suppression, angiogenesis, and metastasis [91]. Adenosine contributes in immunosuppression by modulating CTL and NK cells mediated antitumor immune response, so elevated enzymatic activity of CD73 in exosomes, which also present PD-1 molecules, is thought to reduce the effectiveness of anti-PD-1 agents [93, 94]. In that regard, CD73 or adenosine targeting with monoclonal antibodies for melanoma treatment is being studied [91].

CD39 is also an ectonucleotidase that contributes in adenosine production, alongside CD73. CD39 and CD73 are both overexpressed in cancer cells to induce immune suppression and have been detected in exosomes [95, 96]. Both markers are upregulated in hypoxia and in the presence of several pro-inflammatory molecules, and their presence in several malignancies suggests their role in the progression of neoplasia. CD39/CD73 system is related to tumour growth, angiogenesis, invasion, migration and metastasis, as well as immunevasion by inhibiting the activation, expansion and homing of tumour specific T helper and CTL, which leads to T cell exhaustion [97].

T cell immunoglobulin and mucin domain-containing protein 3 (TIM-3) is a T cell inhibitory receptor that mediates T cell apoptosis upon engagement by its ligand galectin-9. Because of that, high concentrations of galectin-9 are found in metastatic melanoma tumour environment [98]. Co-expression of TIM-3 with PD-1 are signals of exhausted CD8 + T cells, but both CD4 + and CD8 + are susceptible of Gal-9-induced cell death [98]. In that regard, MV3 melanoma cells deliver TIM-3 to CD4 + T cells via exosomes to inhibit their function. Moreover, they also induce macrophage M2 polarisation, which promotes tumour growth and metastasis [99].

Normally, hepatocyte growth factor (HGF)/mesenchymal-epithelial transition factor (c-MET) signalling is involved in embryonic development and tissue regeneration because interaction of both factors avoids apoptosis and stimulates proliferation and motility. Therefore, tumour cells also use its activity to promote tumour progression, but, unlike melanocytes, melanoma cells are able not only to express c-MET, but also to release HGF in an autocrine manner for self-stimulation [100]. Additionally, c-MET oncoprotein on melanoma cell-derived exosomes educates BMDCs toward a pro-vasculogenic and pro-metastatic phenotype [40] and c-MET-low melanoma cells can upregulate c-MET to adapt to its needs, like facilitate metastasis, because it gives cells invasion and motility capacity [101]. Nevertheless, MET inhibitors have currently been studied, which have been proven to inhibit melanoma cell migration [102].

HSP90 (heat shock protein of 90 kDa) is a chaperon involved in proper protein folding and maturation, but also plays a crucial role in cancer cell survival. HSP90 level increases with melanoma progression due to the high proliferation rate, low pH and the need to control proteostasis [103]. Apart from that, HSP90 in melanoma cells is involved in myeloid-derived suppressor cell (MDSC) differentiation, which exert many immunosuppressive functions (e.g. T cell activation inhibition or induction of Treg) [104]. In that regard, HSP90 has been found in melanoma derived extracellular vesicles to educate bone marrow cells and create a metastatic niche [40]. It should be noted that, inhibitors of HSP90 are being tested for melanoma treatment [103].

Finally, some melanoma cells and vesicles have tested positive for the non-classical human leucocyte antigen (HLA)-G class I. HLA-G1 is a key molecule in maternal-foetal tolerance because it binds to inhibitory receptors of activated immune cells, such as NK cells, T cells and APCs. Hence, cancer cells upregulate its expression in order to scape immunosurveillance [105, 106]. Nevertheless, HLA-G1 is not able to induce immune tolerance on its own, and more suppressive factors need to be involved [106].

#### miRNA

microRNA in melanoma-derived exosomes play an important role in tumour progression (Table 1). Exosomes derived from the melanoma cell line A375, for example, are enriched in let7 miRNAs (let-7a, let-7c, let-7f) as well as miR-31, miR34b and miR-185, which favour melanoma metastasis and melanoma invasion, respectively [107].miRNA-222 expressed in EVs increases melanoma malignancy [108] and plasticity [109]. EVs derived from primary melanoma cells proved to express miRNA-222. Through exosomes, melanoma cells effectively transferred the miRNA to acceptor cells and, in consequence, miRNA-222 modulated several processes—such as PI3K/AKT pathway—resulting in cell proliferation, differentiation and apoptosis blockage [108]. Specifically, miRNA-222-5p influences the tumour aggressiveness [109].

Impairing T cell activity is another approach by which miRNAs can promote tumour development. miRNAs present in B16 derived exosomes, including miR-690, activate the mitochondrial apoptotic pathway of CD4 + T cells. The activation of caspase 3, 7 and 9 in CD4 + T cells downregulated the anti-apoptotic proteins and resulted in faster melanoma cell growth in mice. However, they did not induce apoptosis in CD8 + T cells [110].

To impair CD8 + T cell activity, melanoma cell-derived exosomes can decrease T cell receptor (TCR) signalling and TNF-α secretion. Melanoma exosomes enriched in miR-3187-3p, miR-498, miR149 and miR-181a/b are responsible for the downregulation of TCR signalling, probably due to the interaction with CD45 and the resulting loss of its phosphatase activity. In addition, through TCR downregulation and other indirect regulation pathways, TNF-α secretion is altered. Those effects can result in T cell suppression and immune evasion [111].

## Melanoma-derived EVs in therapy

Even though tumour EVs have potential to promote tumour growth and immunosuppression, they can exert anti-cancer activities in immunotherapy. Tumour EVs carry tumour antigens—MHC-I molecules and HSP, among others—and can efficiently deliver them to DCs, which in turn trigger CD8 + T cell dependent anti-tumour responses [112].

The goal of this section is to determine the therapeutic potential of melanoma cell-derived exosomes for melanoma treatment, including non-modified or constitutive EVs, modified EVs and stressed cell derived EVs (Table 2).synthetic bacterial vesicles, SyBV; anti- programmed cell death protein 1, anti-PD-1; streptavidin-lactadherin, SAV-LAV; Glu-Ala-Leu-Ala, GALA; major histocompatibility complex type I, MHC I; class II transcriptional activator, CIITA; MHC type II, MHC II; heat shock protein 60 and 70 kDa, HSP60 and HSP70; dendritic cell, DC; cytosine-guanine DNA, CpG DNA

**Table 2:** Melanoma-derived EVs for therapy. Studies are listed by the order of presentation in the text

First author	EV origin	Modification	Resulting EVs	Adjuvant	Effect	Ref
Muhsin-Sharafaldine et al.	B16	None	Apoptotic EV, microvesicles and exosomes	None	Enhanced anti-tumour immunity responses with apoptotic vesicles than with microvesicles or exosomes	[113]
Kyong-Su Park et al.	B1610 cell line	None		SyBV and anti-PD-1	Enhanced tumour specific humoral and cellular immune responses, and inhibition of tumour growth	[114]
Lee et al.	B16F1	*CIITA* transduction	High levels of antigen-MHC II molecules in exosomes	None	Specific CD4 + and CD8 + populations and prolonged mice survival rate	[115]
Morishita et al.	B16BL6	Transfection with a plasmid vector encoding SAV-LA and biotinylated GALA	pH sensitive exosomes	None	Enhanced antigen-MHC I presentation in DCs	[116]
Morishita et al.	B16BL6	Transfection with a plasmid vector encoding SAV- LA	Exosomes expressing SAV-LA	Biotinylated CpG DNA	Enhanced tumour antigen presentation	[117]
Koyama et al.	B16	*M. tuberculosis* antigen transfection	Exosomes expressing *M. tuberculosis* antigen	Bacterial antigen	Enhanced suppression of tumour growth	[118]
Horrevorts et al.	Mel-JuSo and SK-MEL28	High-mannose glycans	Apoptotic EVs with high-mannose glycans		Tumour-specific CD8 + T cells	[119]
Chen et al.	B16	Heat treatment	Enriched in HSP60 and HSP70	None	Enhanced CD4 + and CD8 + cell attraction	[121]
Kim et al.	B16BL6	Gamma radiation		None	DC maturation, pro-inflammatory cytokine release and antigen-specific T cells	[122]
Komarova et al.	B16	HSP70 treatment	HSP70 enriched exosomes		Enhanced CD8 + response and antitumor cytokine release	[43]
Wang et al.	B16F10	Azide integration	Functionalised exosomes		Not yet tried in vivo nor in vitro	[123]

### Non-modified melanoma EVs

The use of non-modified melanoma cell-derived EVs as tumour vaccines is controversial. Their innate pro-tumour functions can act as double-edged sword, and they need to be understood in order to overcome their pathogenicity. Afterwards, EVs can be administered alongside other formulations to induce the desired immune response.

In a study using B16 melanoma cell line, different EVs were compared to assess the immunogenic properties of each one. Results concluded that apoptotic vesicles provided higher protection compared to exosomes or microvesicles [113]. However, those results surprised the authors because exosomes possess more antigens and immune response stimulating capacity than apoptotic vesicles; the contradiction was probably related to experimental conditions. Nevertheless, melanoma derived EVs lack the immunostimulatory capacity to activate DCs, and so, they have to be administered with an adjuvant or another immunotherapy.

The absence of articles using just melanoma cell-derived EVs to induce an immune response in cancer reflects the low ability of these EVs to induce an adequate immune response on their own. Hence, EVs must be complemented with immunostimulatory strategies.

**Fig. 5:**
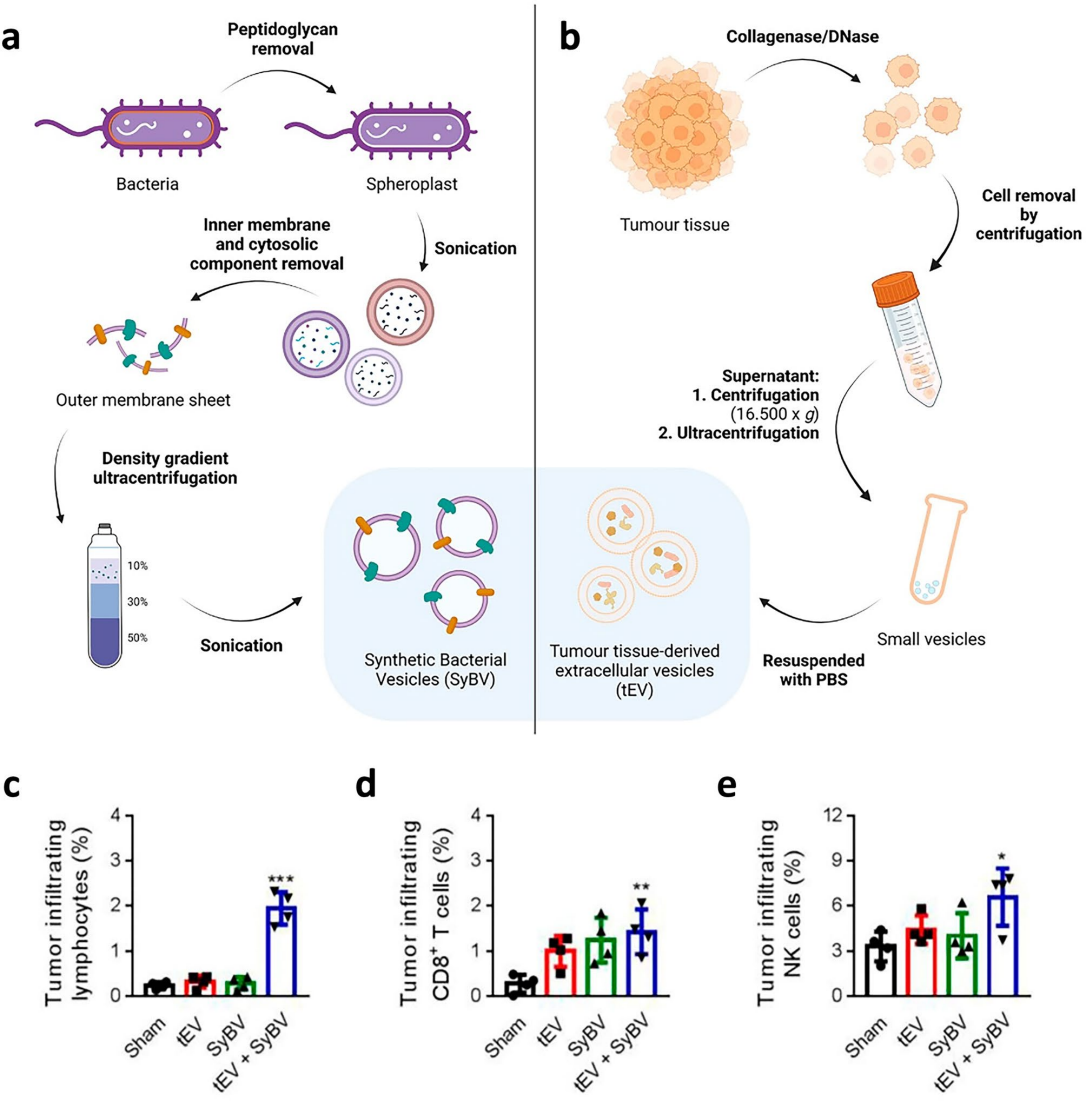
Immunisation with melanoma cell derived EVs (tEVs) combined with synthetic bacterial vesicles (SyBV) induces tumour-specific immunity. **a**, **b** Schematic diagram of SyBV preparation and tEV isolation from tumour tissue.** a** *E. coli* outer membranes were purified from culture. Peptidoglycan was removed and the resulting spheroplasts were pelleted and then sonicated. The unbroken cells were removed by centrifuging, and then whole membranes were pelleted from the supernatants. The membranes were incubated in 0.5% Sarkosyl, and the outer membranes were pelleted. Next, the pellets were incubated with high pH solution, then applied to a iodixanol density gradient in an ultracentrifuge tube (50%, 30% and 10% iodixanol). The layers formed between 10 and 30% iodixanol after ultracentrifugation were collected. Finally, the samples were sonicated for and considered as SyBV. **b** Small tumour pieces from humans or mice were incubated with collagenase D and DNase I to dissolve fibrotic structures. Cells and tissue debris were eliminated by centrifugation. Supernatants were centrifuged and ultracentrifuged to collect large and small vesicles, respectively. Only small vesicles were resuspended in PBS, and these were considered tEV. **c**–**e** Total tumour-infiltrating lymphocytes were analysed by flow cytometry on day 17 following immunisation with tEV and/or SyBV (*n* = 4). **c** Average percentages of cells in tumour tissue. Mean percentages of **d** CD8 + T cells and **e** NK cells. Modified from [114] and created with BioRender.com

In order to promote the immunostimulatory capacity, Kyong-Su Park et al. subcutaneously administered B16F10 melanoma cell line derived EVs alongside synthetic bacterial vesicles (SyBV) (Fig. 5). Melanoma EVs served as cancer antigen source, and SyBV were used to stimulate the immune response and achieve enhanced CTL and Th1 mediated immunity, which elicited tumour regression in melanoma-bearing mice. SyBVs were produced by chemical modification of bacterial (*E. coli*) membrane, a method that increases vesicle obtaining compared to isolation of the natural vesicles secreted by the bacteria (bacterial outer membrane vesicles (OMV)). SyBVs are less toxic than naturally released OMVs, but still able to effectively activate mouse bone marrow-derived dendritic cells. They also compared the results with other immune adjuvants like CpG, IFA and alum, demonstrating the superiority of the developed adjuvant for the combination of immunostimulants naturally presented in bacterial membranes. Moreover, they also compared the results of intraperitoneally injected anti-PD-1 treatment efficacy and concluded that it synergistically enhanced the therapeutic effect, demonstrating the potential immunotherapy combination can have [114].

### Modified melanoma EVs

In another approach, melanoma-derived EVs can be modified to alleviate the negative effects they present, such as lack of immunogenicity or the overexpression of immunomodulatory molecules. As a result, modified EVs can trigger effective anti-tumour responses.

One way to potentiate anti-tumour responses induced by exosomes is enhancing the recognition of antigens in major histocompatibility complexes (MHC). MHC molecules in DCs are necessary for an efficient tumour antigen presentation, and exosomes can be used to achieve that goal: MHC class I molecules to induce cytotoxic CD8^+^ T cells, and MHC class II molecules to effectively activate CD4^+^ T cells. Exosomes with MHC II molecules can facilitate MHC II- melanoma antigen presentation in DCs. B16F1 murine melanoma cell line transducted with *CIITA* (class II transcriptional activator) gene produced exosomes with high levels of MHC II molecules. The melanoma exosomes expressed the MHC II molecules complexed with tumour antigenic peptides. Compared with unmodified exosomes, CIITA-exosomes increased IFN-γ-producing CD4 + T cell and tumour antigen (TYRP2)-specific CD8 + T cell populations, and prolonged survival rate of the tumour-bearing mice [115].

In order to enhance MHC I melanoma antigen presentation, B16BL6 derived melanoma exosomes can be modified with GALA (Glu-Ala-Leu-Ala), a pH-sensitive fusogenic peptide. Melanoma cells transfected with a plasmid vector streptavidin (SAV)-lactadherin (LA) secreted SAV-exosomes, which bound biotinylated-GALA. When DCs endocytose GALA-exosomes, the acidic conditions activate GALA membranolytic capacity and the cargo is released to the cytosol. In consequence, MHC I-tumour antigen complex presentation in DCs is enhanced [116]. Taken together, exosomes that potentiate antigen presentation in MHC molecules can be an interesting strategy in immunotherapy.

**Fig. 6:**
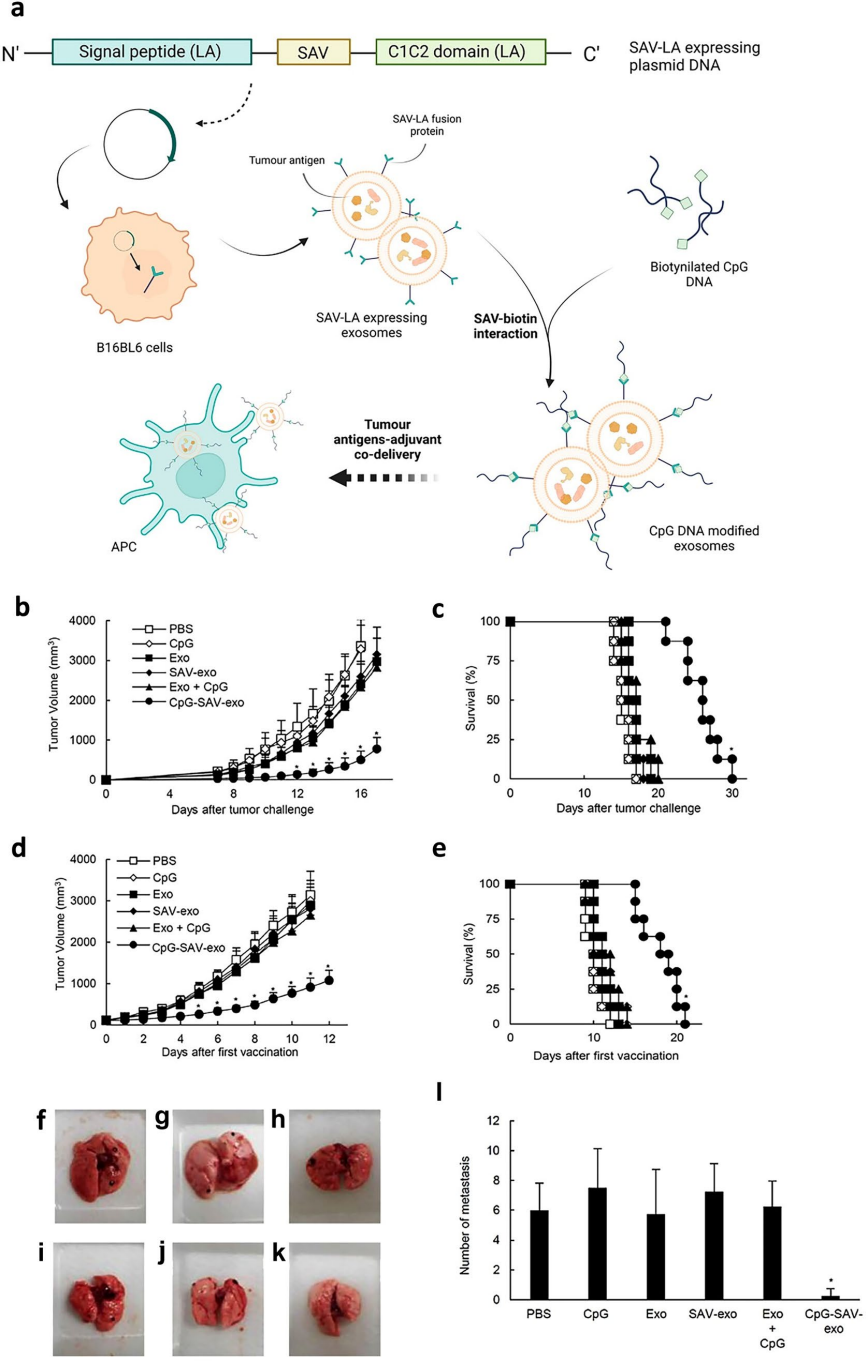
Melanoma cell-derived exosomes with immunostimulatory CpG DNA. **a** Schematic representation of the preparation of CpG DNA-modified exosomes. A plasmid DNA encoding a fusion protein of streptavidin (SAV), N-terminal secretion signal of lactadherin (LA) and C1C2 domain of LA (SAV-LA) was constructed. SAV-LA expressing exosomes (SAV-exo) were collected from the culture supernatants of B16BL6 cells transfected with the plasmid DNA. CpG DNA-modified exosomes (CpG SAV- exo) were prepared by mixing SAV-exo and biotinylated CpG DNA. **b**–**e** Two approaches were tested: immunitation pre- or post-tumour development. **b**, **c** Mice were intradermally immunised three times, at 3-day intervals, with the following formulations: PBS, CpG-SAV-exo, SAV-exo, Exo-CpG, or CpG-SAV-exo (1 pmol DNA and 1 µg exosomes in 50 µL PBS per mouse). At day 7 post-immunisation, mice were subcutaneously inoculated with B16BL6 cells (5 × 10^5^ cells/mouse). **d**, **e** Mice were subcutaneously inoculated with B16BL6 cells to induce tumour development. When tumour volume exceeded 100 mm^3^, formulations were directly injected into tumours, at 3-day intervals. Graphics show daily measured values of **b**, **d** tumour volume and **c**, **e** mice survival. Results are expressed as the means ± standard deviations (*n* = 8). Data shown are representative of two independent experiments. **f**–**l** The effect of the formulations in pulmonary metastasis was measured. B16BL6 cells were inoculated to mice, and then they were treated. At 1 day after the last immunisation, lungs were isolated and the number of pulmonary tumour nodules were counted. Photographs of lungs after the treatment with **f** phosphate-buffered saline (PBS), **g** CpG, **h** exosome (Exo), **i** SAV-LA-expressing exo (SAV-exo), **j** Exo- CpG, and **k** CpG DNA-modified exo (CpG-SAV-exo). **l** Number of pulmonary tumour nodules. Results are expressed as the means ± standard deviations (*n* = 4). *P < 0.05 compared with the Exo-CpG group. Adapted from [117] and created with BioRender.com

EVs can also be engineered to express in the surface an immune stimulant. In that way, antigens naturally presented in EVs will be delivered alongside the immune stimulant DCs need for a proper maturation and antigen presentation. With the same technology used for the previously explained GALA modification, Morishita M. et al. modified B16BL6 murine melanoma cell line to attach the Toll-Like Receptor 9 agonist (TLR9) CpG DNA to exosomes (Fig. 6). The intratumoural injection of CpG DNA-modified exosomes (CpG-SAV-exo) in a mouse model exerted stronger antitumor effect than the simple administration of both components [117].

Not only TLR agonist but also antigens that our immune response already recognise can be an interesting approach in order to activate the immune system. For that end, B16 melanoma cells were transfected with a *Mycobacterium tuberculosis* antigen. Mice injected with modified exosomes developed immunity against both bacterial and tumour antigens. Moreover, intratumoural injection of modified exosomes significantly suppressed tumour growth compared to unmodified ones [118].

Another study demonstrated that if melanoma cells (Mel-JuSo and SK-MEL28) are modified to express high-mannose glycans, apoptotic tumour cell-derived extracellular vesicles (ApoEVs) will express them too after apoptosis induction. High-mannose glycans are ligands for a DC receptor, which efficiently internalises the cargo to the cell and to both MHCs. Results showed that modified ApoEVs enhanced priming of tumour-specific CD8 + T cells [119].

### Stressed cell derived melanoma EVs

Finally, EVs can be modified without cell transfection. The last way proven to potentiate the immune activity of melanoma-derived EVs is by heat treatment of the tumour cells. Tumour cell derived EVs secreted under heat can be more effective in activating immune cells towards an antitumour effect as they are enriched in tumour antigens and heat shock proteins like HSP60 and HSP70 [120]. In an study carried out with a Lewis lung carcinoma 3LL cell line and B16 melanoma cell line, heat-stressed tumour cells (HS-TEX) contained CL2, CCL3, CCL4, CCL5 and CCL20 chemokines and were able to attract CD11c( +) DC, CD4( +) and CD8( +) T cells. This means that intratumoural administration of HS-TEX inhibits tumour growth and prolongs survival in vivo [121].

Not only heat-treated but also gamma-irradiated cells also produce more immunogenic exosomes that non-irradiated ones. In a study using gamma irradiation, exosome production in melanoma cells was increased after the treatment, and the exosomes entered in DCs more easily due to a higher binding capacity. In addition, gamma irradiation increased the damage-associated molecular pattern (DAMP) protein HMGB1 (high mobility group box-1). As a result, irradiated exosomes induced better maturation of DCs than non-irradiated ones, and also promoted T cell proliferation and generation of T helper type 1 (Th1) and interferon (IFN)-γ-producing CD8^+^ T cells. In the study, exosomes were used to pulse DCs, which were then administered in a mouse melanoma model. Results showed a higher frequency in tumour antigen-specific T cells in the irradiated group [122].

HSP70 is an immunostimulatory chaperone, discussed in the previous section, with which EVs can be enriched. Recombinant HSP70 treatment in melanoma B16 cells triggered EV mediated release of its intracellular analogue in a study carried out by Komarova, E.Y. et al. [43]. The resulting EVs were subcutaneously administered alongside melanoma cells in mice. HSP70-high EVs induced a potent anti-cancer response in vivo, with CD8^+^ cell response and anti-tumour cytokine accumulation. In addition, delay in tumour growth and longer survival period were registered. Moreover, the EVs also inhibited the maturation of pro-tumour macrophages. These results show a promising EV modification technic as it is easy to carry out and provoke an effective immune response.

B16F10 melanoma cells cultured in the presence of L-azidohomoalanine (AHA) and tetraacetylated N-azidoacetyl-D-mannosamine (ManNAz) to produce azide-integrated exosomes, combined with biorthogonal click conjugation, allows the functionalisation of exosomes with a wide variety of molecules for intracellular delivery [123]. Although this approach has not been yet tried in vitro nor in vivo, it shows the functionalizing capacity of exosomes.

Modifications of EVs show great promise, but there are many EV modification techniques that have not been yet tried for melanoma EVs. In that regard, there is plenty of room to continue investigating in melanoma EV engineering.

## Melanoma cell membrane as antigen source

Because cancer cell membranes have many tumour-related proteins, they can act as cancer antigen source for immunotherapy, just like cancer-derived EVs. Vesicles can be produced from tumours by homogenisation of the tissue [124, 125] or apoptosis induction [119], and still contain TSA and TAA. With this approach, EV vaccine scalability issues can be overcome. Furthermore, melanoma membrane can be modified to further express adjuvants for immune stimulation and be carried by particles that are also able to exert immune response (Table 3).polyinosinic:polycytidylic acid, Poly(I:C); polietilenglicol, PEG; nanoparticle, NP; programmed cell death protein 1, anti-PD-1; cytotoxic T lymphocyte, CTL; bacterial outer membrane, OMV; hollow polydopamine, HDPA; near infrared, NIR; monophosphoryl-lipid A, MPLA; poly(lactic-co-glycolic acid), PLGA; microvesicle, MS; interleukin-6, IL-6; tumour necrosis factor alpha, TNF-α

**Table 3:** Melanoma cell membrane particles. Studies are listed by the order of presentation in the text

Author	Melanoma cell source	Modification	Resulting EVs	Adjuvant	Effect	Ref.
Lee et al.	Melanoma tissue	Homogenisation and sonication	Nanovesicles	Poly(I:C)	Reduced number and size of metastasis	[125]
Ochyl et al.	B16F10 OVA	Lysis, sonication, aggregation and PEGylation	PEG-NPs	Anti-PD-1	Strong CTL response and regression of tumours in animals	[124]
Wang et al.	B16-F10	OMV fusion and HPDA encapsulation	NIR activated melanoma-OMV particles	NIR laser radiation	Immune response activation and tumour eradication	[126]
Jung et al.	A549 and B16F10	Disrupted, sonicated, and modified to express an azide group	MPLA encapsulated PLGA MSs coated with melanoma cell membrane	MPLA	In vitro IL-6 and TNF-α release	[127]
Yang et al.	B16-OVA	Mannose modification of the membrane	PLGA NPs loaded with R837, and coated with mannose modified melanoma membrane	R837 and anti-PD-1	DC maturation, specific CTL response and enhanced mice survival	[128]

In a study that homogenised and sonicated autologous melanoma tissue for nanovesicle (NV) production, vesicular antigens were increased 30–40-fold compared to naturally produced exosomes. The NVs, alongside poly(I:C) as adjuvant, were able to reduce the number and size of the metastasised colonies in mouse melanoma models [125]. Nevertheless, the NVs were not compared to exosomes and, in consequence, the anti-tumour effect of both must be compared in order to conclude their suitability.

**Fig. 7:**
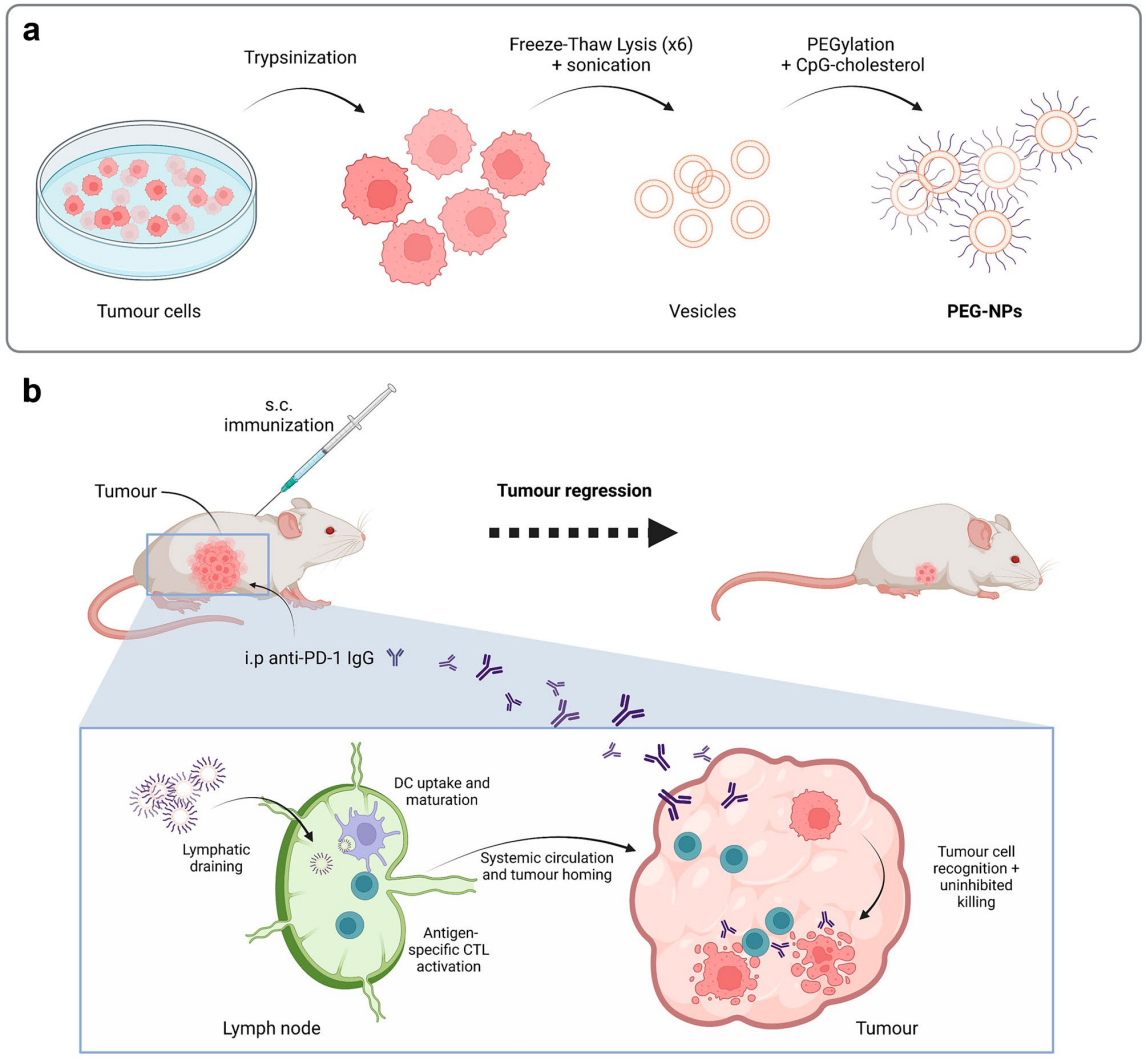
Schematic representation of PEG-NPs preparation and therapy. **a** B16F10 OVA cells are lysed via freeze–thaw cycling, sonicated to form nano-sized vesicles, collected after calcium-mediated aggregation and washed. PEGylation, removal of calcium with EDTA, and further wash steps are then performed, resulting in the formation of PEG-NPs. Finally, for mice immunisations, cholesterol-linked CpG is incorporated to nanoparticles by incubating both for 30 min at 37 °C. **b** Upon subcutaneous administration in tumour-bearing mice, PEG-NPs drain efficiently to lymph nodes where they are taken up by DCs for activation of antigen-specific cytotoxic CD8 + T lymphocytes (CTLs). After tumour-infiltration, CTLs recognise and kill cancer cells in synergy with anti-PD-1 immune checkpoint blockade, leading to tumour regression. Adapted from [124] and created with BioRender.com

Ochyl, L. J. et al. lysed and sonicated B16F10 OVA (ovalbumin) cells, and then aggregated them to form membrane particles. The resulting nanoparticles (NPs) were PEGylated to form PEG-NPs. For in vivo immunisations, cholesterol-modified CpG was added to the PEG-NPs (Fig. 7). Strong CTL responses were obtained in vivo, especially in combination with anti-PD-1 therapy, which resulted in complete regression of tumours in 63% of the animals [124].

In another line, melanoma cell membrane can be modified as well as used to coat particles. A hybrid membrane formed with B16-F10 cancer cell line (CC) and bacterial outer membrane (OMV) was developed to combine homing ability and immune activation properties of both. The resulting membrane was then used to encapsulate hollow polydopamine (HPDA) NPs, a photothermal agent. Results showed that i.v. administration of the developed NPs activated immune response and accumulated in tumour area; moreover, under NIR laser irradiation the tumour disappeared in over 80% of the mice [126].

Jung, H. et al. developed a PLGA microsphere (MS) coated with cancer cell-derived membrane vesicles (VE) as antigen source. Cells from A549 and B16F10 cell lines were used for vesicle generation. First, the membrane of the cells was disrupted and sonicated to generate vesicles and modified to present an azide group. Then, PLGA microspheres were prepared by double emulsion and solvent evaporation method, which enabled monophosphoryl-lipid A (MPLA) encapsulation and modified to express alkyne groups. Finally, both formulations were stuck together via click chemistry. They demonstrated that MS-VE was efficiently taken up and triggered the in vitro release of immune stimulating cytokines, IL-6 and TNF-α [127].

Lastly, membrane-coated particles can be further modified; for example NPs with mannose-modified tumour cell membranes. Yang R. et al. loaded PLGA NPs with R837, a toll-like receptor 7 (TLR-7) agonist, and then they used membranes from B16-OVA cancer cells to encapsulate the NPs. The obtained NPs were further modified with mannose by a lipid-anchoring method. Nanoparticles showed maturation of DC and specific CTL response to B16OVA cancer cells, leading to immune protection with prophylactic capacity. Furthermore, developed NPs combined with anti-PD-1 showed statistically relevant mice survival rate, compared to anti-PD-1 or NPs alone [128].

## Conclusions and future prospects

EVs are the hotspot in cancer research, and in the last years, melanoma-derived EVs function and content has been thoroughly studied. Many of the functions conferred to melanoma cells are replicated by their EVs, such as immunomodulatory capacity, but they also possess tumour-related antigens and immunostimulatory molecules expressed in their parent cell. Thus, correct characterisation of and further molecule identification in EVs will give us key information about the development of melanoma, but also its weaknesses.

To date, most of the research carried out concerns just exosomes, and studies regarding other EVs and their contribution in melanoma and its treatment is needed. In any case, this is still quite difficult as isolation technics need to be further improved, or, at least, standardised technics should be implemented to allow consistent isolation of EVs. Moreover, experimental conditions should also be standardised and optimised as they have great impact in cells. Only then, EV subpopulation heterogeneity, generation processes and functions will be properly described and understood.

Regarding patient-derived EVs, identifying their origin and molecular cargo, as well as their effect on acceptor cells might help us know whether the patient is prone to receive immunotherapy or its EVs can act as tumour antigen carriers. Nevertheless, more studies carried out with EVs derived from melanoma patients should be performed to develop feasible and representative therapies, as most of them use melanoma cell line derived EVs.

EVs present many possible uses as they are the reflexion of the parent cell. EVs as antigen source for cancer vaccine are very interesting option because they express TSA and TAA from tumour cells that the immune system recognises. Moreover, there is no need for biopsies to determine antigens expressed in the tumour as EVs can be obtained from blood samples.

Nonetheless, in order to use EVs as antigen source we have to approach some issues. First, we have to optimise EV isolation techniques, especially the ones obtained from patients’ blood to acquire a high number of EVs with optimal purity. Secondly, cancer-derived EVs show many immunomodulatory molecules that hinders the activity of the immune cells, so immunosuppressive effects need to be blocked. In that regard, immunostimulatory effects have to counteract immunosuppressive ones, whether combining EVs with immunoadjuvants or blocking EVs’ immunosuppressive effects.

Taken together, EVs are a potent tool for cancer research and therapy. Yet, most research focuses on their capacity to promote melanoma but evade immunosupportive ones. Future investigation should assess their dual effect, and the strategies we should develop to direct their potential effect to our interest: cancer treatment.

## Data Availability

Not applicable.
